# Data on exercise and cardiac imaging in a patient cohort with hypertrophic cardiomyopathy

**DOI:** 10.1016/j.dib.2017.08.018

**Published:** 2017-09-12

**Authors:** Lars A. Dejgaard, Trine F. Haland, Oyvind H. Lie, Margareth Ribe, Thea Bjune, Ida Skrinde Leren, Knut Erik Berge, Thor Edvardsen, Kristina H. Haugaa

**Affiliations:** a Department of Cardiology and Center for Cardiological Innovation, Oslo University Hospital, Rikshospitalet, Oslo, Norway; b Institute for Clinical Medicine, University of Oslo, Oslo, Norway; c Institute for Surgical Research, Oslo University Hospital, Rikshospitalet, Oslo, Norway; d Unit for Cardiac and Cardiovascular Genetics, Department of Medical Genetics, Oslo University Hospital, Oslo, Norway

**Keywords:** Hypertrophic cardiomyopathy, Exercise, Genetics, Arrhythmia

## Abstract

Data presented in this paper are supplementary material to our study “Vigorous exercise in patients with hypertrophic cardiomyopathy” [1]. The current article presents supplementary data on collection and analyses of exercise parameters and genetic data in the original research article.

**Specifications Table**

**Table:** 

Subject area	*Medicine*
More specific subject area	*Cardiology- cardiomyopathies*
Type of data	*Tables, images, questionnaire*
How data was acquired	*Survey (physical activity questionnaire), Echocardiography (Vivid 7 and Vivid E9 - GE Healthcare, Horten, Norway), Cardiac magnetic resonance (Magnetom Sonata and Magnetom Avanto Siemens, Erlangen, Germany), Holter*
Data format	*Analyzed and raw material*
Experimental factors	*Statistical analysis with SPSS version 21.0, SPSS Inc., Chicago, IL, USA.*
Experimental features	*Cross-sectional population study*
Data source location	*Oslo, Norway*
Data accessibility	*Data is with this article*

**Value of the data**

• Extensive clinical information and imaging data on study subjects.
• Extensive information on exercise habits and clinical endpoints in our hypertrophic cardiomyopathy study cohort.
• Physical exercise questionnaire used in the survey
• Complete genetic information on all mutations in our hypertrophic cardiomyopathy study cohort, with potential for pooling of data and study mutation-specific variations in phenotypic expression.

## 1 Data

The data presented in this article are supplementary material to our study on vigorous exercise in hypertrophic cardiomyopathy patients. [1].

**Fig. A.1:**
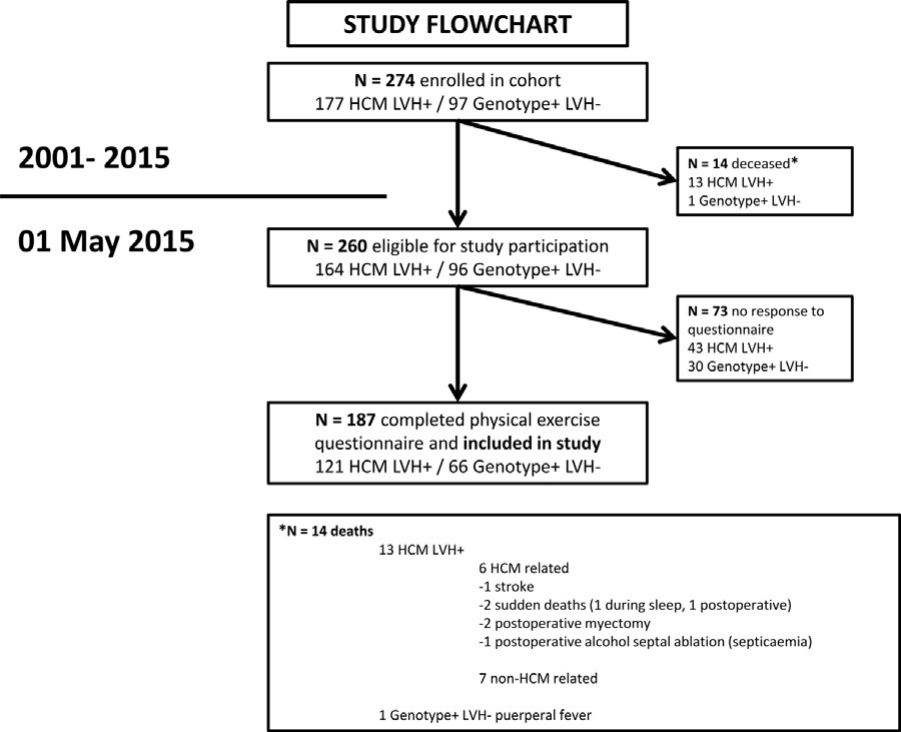
Study flow chart. HCM LVH+=Hypertrophic cardiomyopathy phenotype positive; Genotype+ LVH-=Hypertrophic cardiomyopathy genotype positive, phenotype negative.

Fig. A.1 displays study flowchart and A.2 and A.3 represent the letter and the physical activity questionnaire sent to study participants. Tables B.1 and B.2 are logistic regression models presenting markers of hypertrophic cardiomyopathy phenotype and ventricular arrhythmias. Table B.3 presents specific information on genetic mutations. Table B.4 contains clinical and imaging data related to hypertrophic cardiomyopathy phenotype and exercise status. Table B.5 shows additional exercise data.

## 2 Experimental design, materials and methods

The study design was cross-sectional. Time of study inclusion was the first clinical evaluation and echocardiogram in the outpatient clinic, Unit for Genetic Cardiac Diseases, Department of Cardiology, Oslo University Hospital, Rikshospitalet, Norway (Fig. A.1). Hypertrophic cardiomyopathy genotype positive, phenotype negative (Genotype+ LVH-) and hypertrophic cardiomyopathy phenotype positive (HCM LVH+) patients were included. Inclusion commenced in 2001 and ended in 2015 [1].

In May 2015 we cross checked the HCM cohort against the Norwegian death registry and found 14 deaths, of which cause of death was documented for all cases in the medical journals, and is reproduced in Fig. A.1. During May 2015 we completed a physical activity survey via letter (A.2 and A.3) among the 260 live subjects enrolled in our HCM cohort. Non-responders were contacted by phone and offered the possibility of completing the physical activity questionnaire via structured interview (A.3).

Study participants who were actively participating in organized or competitive sports at study inclusion, were defined as competitive athletes.

## Funding sources

This work was supported by the Norwegian Research Council [203489/030].

## Figures and Tables

**Fig. A.2:**
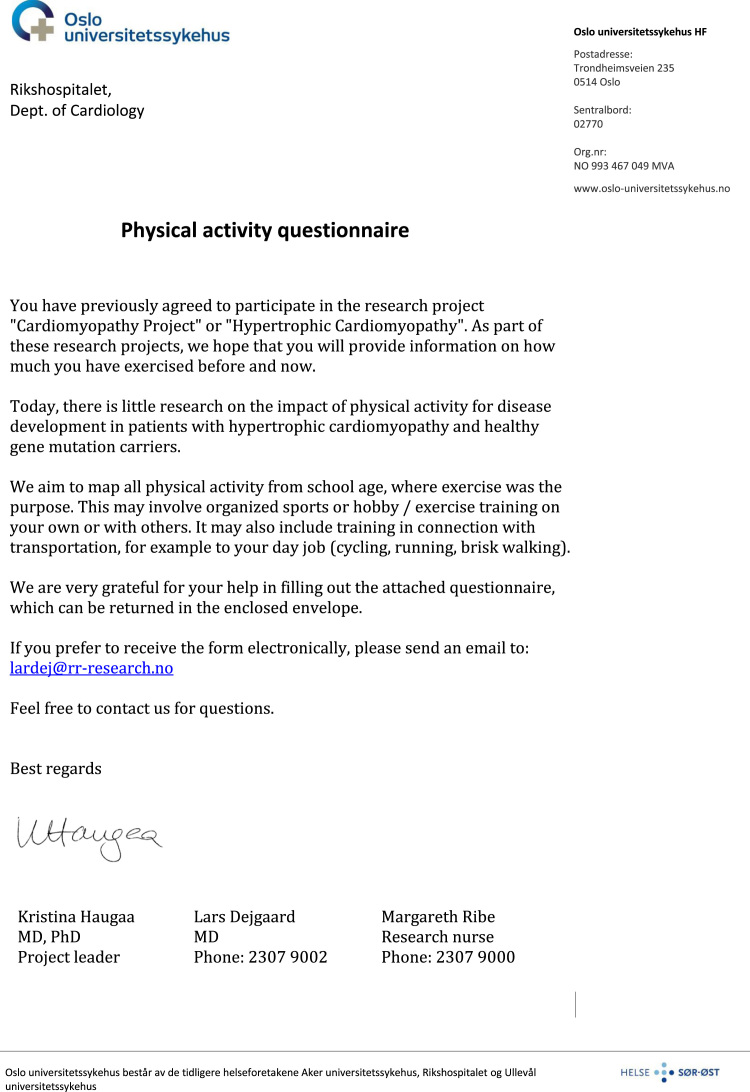
Letter sent to study participants together with physical activity questionnaire (A.3). Translated from Norwegian to English by the authors.

**Fig. A.3:**
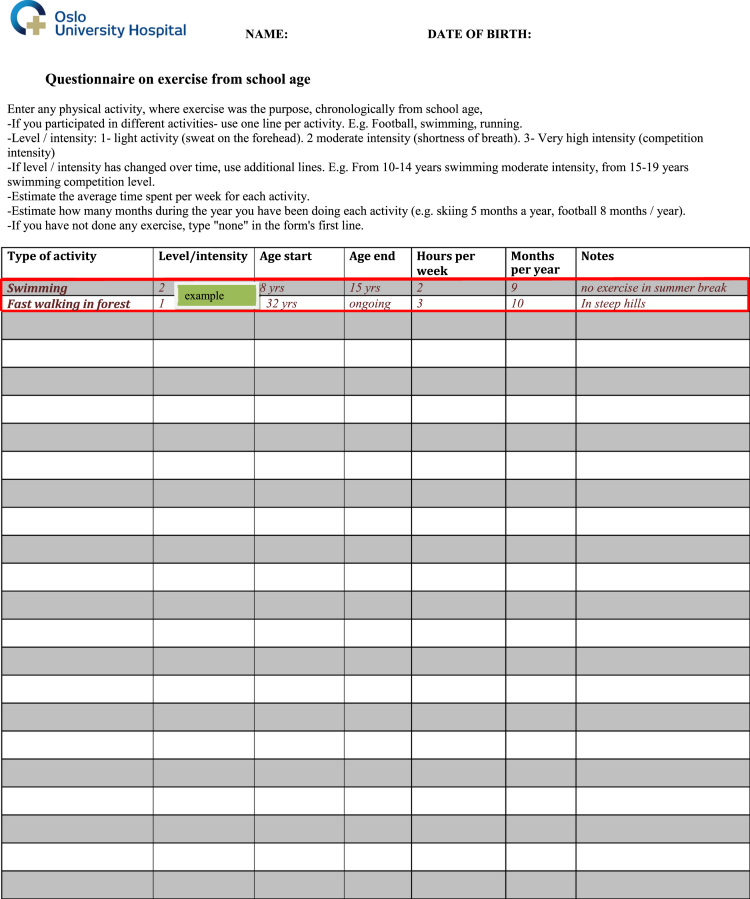
Physical activity questionnaire used in our survey. Translated from Norwegian to English by the authors.

**Table B.1:** Markers of hypertrophic cardiomyopathy phenotype positive (HCM LVH+) (n= 121) in 187 study participants.

***Markers of HCM LVH+ (n= 121) in 187 study participants***				
	**Univariate logistic regression**		**Multivariate logistic regression**	
	**OR, 95% CI**	**P**	**OR, 95% CI**	**P**
Athlete (yes vs. no)	0.78 (0.42–1.43)	0.42	0.73 (0.32–1.67)	0.46
Age (years)	1.08 (1.05–1.11)	<0.001	1.07 (1.05–1.11)	<0.001
Body mass index (kg/m^2^)	1.17 (1.07–1.28)	0.001	1.06 (0.97–1.17)	0.20
Gender (male vs. female)	2.76 (1.48–5.13)	0.001	4.17 (1.85–9.38)	0.001

Values are odds ratio (OR) and p-values by univariate and multivariate logistic regression. CI=confidence interval; HCM LVH+=hypertrophic cardiomyopathy phenotype positive.

**Table B.2:** Markers of ventricular arrhythmia (n=28) in 121 hypertrophic cardiomyopathy phenotype positive.

***Markers of ventricular arrhythmia (n=28) in 121 HCM LVH+***				
	**Univariate logistic regression**		**Multivariate logistic regression**	
	**OR, 95% CI**	**P**	**OR, 95% CI**	**P**
Age (years)	0.99 (0.96–1.02)	0.34		
Athlete (yes vs. no)	0.85 (0.36–2.03)	0.71	0.72 (0.29–1.83)	0.50
Body mass index (kg/m²)	1.02 (0.92–1.13)	0.68		
Gender (male vs. female)	1.24 (0.53–2.93)	0.62		

**Echocardiography**				
Ejection fraction (%)	0.93 (0.87–0.99)	0.03		
Global longitudinal strain (%)	1.21 (1.06–1.38)	0.005	1.16 (0.98–1.36)	0.08
Left atrium diameter (mm)	0.96 (0.91–1.01)	0.14		
Left atrium area index (cm²/m²)	0.91 (0.79–1.04)	0.15		
Left ventricular maximal wall thickness (mm)	1.16 (1.05–1.30)	0.006	1.08 (0.95–1.23)	0.23
LVOT peak gradient (mmHg)	0.99 (0.97–1.00)	0.07		
LVOT peak gradient ≥50 mmHg (yes vs. no)	3.3 (0.93–12.00)	0.07		

Values are odds ratio (OR) and p-values by univariate and multivariate logistic regression. CI=confidence interval; HCM LVH+=hypertrophic cardiomyopathy phenotype positive; LVOT=left ventricular outlet tract.

**Table B.3:** Frequency of sarcomeric mutations (n=127) in the study cohort (n=187).

***Sarcomeric mutation***	***Ref.seq***	***Amino Acid***	***c.DNA***	***Genomic_ref. data***	***rs***	***Affected individuals***
MYBPC3	NM_000256.3	Splice mutation	c.3190+2T>G	Chr11(GRCh37):g.47355106A>C	rs113358486	21
MYBPC3	NM_000256.3	p.W792Vfs*41	c.2373dupG	Chr11(GRCh37):g.47359281dup	rs397515963	21
MYH7	NM_000257.2	p.R1420W	c.4258C>T	Chr14(GRCh37):g.23886807G>A	rs145213771	15
MYL2	NM_000432.3	p.R58*	c.172C>T	Chr12(GRCh37):g.111352092G>A	rs756671869	6
MYBPC3	NM_000256.3	p.D770N	c.2308G>A	Chr11(GRCh37):g.47360071C>T	rs36211723	5
MYBPC3	NM_000256.3	p.R502W	c.1504C>T	Chr11(GRCh37):g.47364249G>A	rs375882485	5
MYL3	NM_000258.2	p.A57D	c.170C>A	Chr3(GRCh37):g.46902303G>T	rs139794067	5
MYBPC3	NM_000256.3	p.G1249V	c.3746G>T	Chr11(GRCh37):g.47353691C>A	rs727504259	3
MYBPC3	NM_000256.3	p.E611K	c.1831G>A	Chr11(GRCh37):g.47362755C>T	rs730880555	3
MYH7	NM_000257.2	p.R442C	c.1324C>T	Chr14(GRCh37):g.23898247G>A	rs148808089	3
MYBPC3	NM_000256.3	p.W965*	c.965G>A	Chr11(GRCh37):g.47367883C>		2
MYBPC3	NM_000256.3	p.F271*	c.812_821+12del	Chr11(GRCh37):g.47369396_47369417del		2
MYBPC3	NM_000256.3	p.K185Wfs*12	c.553-562del	Chr11(GRCh37):g.47371417_47371426del		2
MYBPC3	NM_000256.3	p.Q969*	c.2905C>T	Chr11(GRCh37):g.47356593G>A	rs397515992	2
MYBPC3	NM_000256.3	p.Y548Profs*19	c.1641_1642del	Chr11(GRCh37):g.47363690_47363691del	rs398123279	2
MYBPC3	NM_000256.3	p.I49del	c.146_148del	Chr11(GRCh37):g.47372934_47372936del	rs781207661	2
MYH7	NM_000257.2	p.T1377M	c.4130C>T	Chr14(GRCh37):g.23887458G>A	rs397516201	2
MYH7	NM_000257.2	p.V606M	c.1816G>A	Chr14(GRCh37):g.23896866C>T	rs121913627	2
MYH7	NM_000257.2	p.D554E	c.1662C>A	Chr14(GRCh37):g.23897020G>T	rs750828477	2
TINNI3	NM_000363.4	p.D196N	c.586G>A	Chr19(GRCh37):g.55663249C>T	rs104894727	2
MYBPC3	NM_000256.3	p.Y237S	c.710A>C	Chr11(GRCh37):g.47370037T>G	rs397516070	1
MYBPC3	NM_000256.3	p.L1238P	c.3713T>C	Chr11(GRCh37):g.47353724A>G	rs730880702	1
MYBPC3	NM_000256.3	p.Q998E	c.2992C>G	Chr11(GRCh37):g.47355475G>C	rs11570112	1
MYBPC3	NM_000256.3	p.P955Rfs*95)	c.2864_2865del	Chr11(GRCh37):g.47356633_47356634del	rs397515990	1
MYBPC3	NM_000256.3	p.Y816*	c.2448C>A	Chr11(GRCh37):g.47359096G>		1
MYBPC3	NM_000256.3	p.Y79*	c.237C>G	Chr11(GRCh37):g.47372845G>C	rs730880698	1
MYH7	NM_000257.2	p.S1924Afs*9	c.5769delG	Chr14(GRCh37):g.23882989del		1
MYH7	NM_000257.2	p.R1818W	c.5452C>T	Chr14(GRCh37):g.23884311G>A	rs763073072	1
MYH7	NM_000257.2	p.R1696Q	c.5090G>A	Chr14(GRCh37):g.23884905C>T	rs766831916	1
MYH7	NM_000257.2	p.R1677H	c.5030G>A	Chr14(GRCh37):g.23884965C>T	rs730880914	1
MYH7	NM_000257.2	p.M982T	c.2945T>C	Chr14(GRCh37):g.23892910A>G	rs145532615	1
MYH7	NM_000257.2	p.I913_Q914delinsK	c.2738del	Chr14(GRCh37):g.23893298_23893300del		1
MYH7	NM_000257.2	p.R858C	c.2572C>T	Chr14(GRCh37):g.23894085G>A	rs2754158	1
MYH7	NM_000257.2	p.M849W	c.2546T>C	Chr14(GRCh37):g.23894111A>G	rs397516156	1
MYH7	NM_000257.2	p.A797T	c.2389G>A	Chr14(GRCh37):g.23894525C>T	rs3218716	1
TINNI3	NM_000363.4	p.V147L	c.439G>C	Chr19(GRCh37):g.55665508C>G	rs777782551	1
TNNI3	NM_000363.4	p.A157V	c.470C>T	Chr19(GRCh37):g.55665477G>A	rs397516353	1
TNNT2	NM_001001430.2	p.R278H	c.833G>A	Chr1(GRCh37):g.201328372C>T	rs397516484	1
TNNT2	NM_001001430.2	p.K253R	c.758A>G	Chr1(GRCh37):g.201330429T>C	rs3730238	1
TNNT2	NM_001001430.2	p.E195K	c.583G>A	Chr1(GRCh37):g.201331147C>T	rs150008205	1

**Table B.4:** Clinical and cardiac imaging characteristics of 187 study participants, grouped according to phenotype and exercise status (competitive athletes vs. subjects not fulfilling competitive athlete definition).

		***Genotype+LVH−(n= 66)***		***HCM LVH+(n=121)***	
		**Not fulfilling definition of competitive athlete (n=50)**	**Competitive athlete (n=16)**	**Not fulfilling definition of competitive athlete (n=110)**	**Competitive athlete (n=11)**
Age, years		41 ± 15	27 ± 10	55 ± 14	48 ± 15
Atrial fibrillation, n (%)		0	0	19 (17)	0
Betablocker therapy, n (%)		8 (16)	0	89 (81)	7 (64)
Body mass index, kg/m²		24 ± 4.4	23 ± 2.8	27 ± 4	25 ± 4
Female, n (%)		33 (66)	9 (56)	45 (41)	2 (18)
Hypertension, n (%)		3 (6)	0	12 (11)	0
Implantable cardiac defibrillator, n (%)		0	0	15 (14)	1 (9)
	Primary prevention, n (%)			12 (80)	1 (100)
	Secondary prevention, n (%)			3 (20)	0
				
Lifetime vigorous exercise, hours		1396 (0- 10208)	5706 (2689- 10384)	1464 (0- 35776)	7940 (2527- 13993)
Sarcomere protein mutation, n (%)		50 (100)	16 (100)	53 (48)	8 (73)
Ventricular arrhythmias, n (%)		0	0	25 (23)	3 (27)
	Cardiac arrest, n (%)			3 (3)	0
	NSVT, n (%)			22 (20)	3 (27)
				
Vigorous exercise age 7- 20 years, hours		999 (0- 8752)	3400 (65- 8663)	549 (0- 10067)	1986 (728- 8489)
Vigorously exercising at study inclusion, n (%)		20 (40)	16 (100)	23 (21)	11 (100)
				
**Echocardiography**					
E/A		1.5 ± 0.6	1.9 ± 0.5	1.3 ± 0.7	1.5 ± 0.6
e', cm/s		10.8 ± 0.3	14.4 ± 0.2	5.9 ± 3	8.5 ± 3
E/e'		7.0 ± 2.6	5.4 ± 0.9	15 ± 9	10 ± 6
Ejection fraction, %		61 ± 6	58 ± 4	62 ± 7	60 ± 5
Global longitudinal strain, %		-21.5 ± 2.2	-21.2 ± 2.3	-16.5 ± 3.5	-17.6 ± 3.2
Interventricular septal diameter, mm		8.5 ± 2	8.4 ± 1	16.9 ± 0.4	14.4 ± 0.4
Left atrium diameter, mm		34 ± 6	34 ± 3	43 ± 8	40 ± 8
Left atrium area index, cm²/m²		8.7 ± 3	9.5 ± 2	12.5 ± 3	12.0 ± 3
LV end-diastolic diameter, mm		48 ± 4	51 ± 3	47 ± 6	51 ± 6
LV end-diastolic diameter index, mm/m²		27 ± 3	27 ± 2	24 ± 4	26 ± 3
LV end-diastolic volume, cm³		85 ± 26	118 ± 29	81 ± 29	103 ± 40
LV end-diastolic volume index, cm³/m²		47 ± 12	60 ± 13	41 ± 13	52 ± 16
LV end-systolic diameter, mm		31 ± 4	34 ± 4	28 ± 6	32 ± 5
LV end-systolic diameter index, mm/m²		17 ± 2	18 ± 2	15 ± 3	16 ± 1
LV end-systolic volume, cm³		34 ± 11	50 ± 16	31 ± 14	40 ± 14
LV end-systolic volume index, cm³/m²		19 ± 5	25 ± 7	16 ± 6	22 ± 7
LV mass, g		131 ± 34	142 ± 30	255 ± 91	262 ± 106
LV mass index, g/m²		72 ± 16	73 ± 13	132 ± 46	130 ± 41
LV posterior wall diameter, mm		7.7 ± 1	7.6 ± 1	10.2 ± 2	10.4 ± 2
LVOT max gradient, rest/Valsalva, mmHg				14 (2- 128)	4 (2- 47)
LVOT max gradient, stress-echo, mmHg §				56 (4- 166)	41 (16- 65)
LVOT max gradient ≥ 50 mmHg, n (%)		0	0	44 (40)	1 (9)
Maximal wall thickness, mm		8.6	8.6	19.1	16.4
Mitral regurgitation, n (%)		14 (28)	1 (6)	76 (69)	3 (27)
	Mild regurgitation, n (%)	14 (100)	1 (100)	46 (61)	3 (100)
	Moderate regurgitation, n (%)	0	0	26 (34)	0
	Severe regurgitation, n (%)	0	0	4 (5)	0
				
Stroke volume, cm³		52 ± 17	68 ±17	50 ± 17	64 ± 27
Stroke volume index, cm³/m²		29 ± 8	35 ± 8	25 ± 8	31 ± 12
**Cardiac magnetic resonance, n (%)**		**n=0**	**n=0**	**n=69 (63)**	**n=7 (64)**
Ejection fraction, %				70 ± 9	61 ± 6
LGE, n (%) #				41 (60)	4 (57)
LGE, % of LV mass #				0 (0- 23)	0 (0-17)
LV mass, g				231 ± 93	198 ± 72
LV mass index, g/m²				119 ± 46	97 ± 29
Maximal wall thickness, mm				22.6 ± 6	17.8 ± 3

Values are mean ± SD or n (%) or median (range). Genotype+ LVH-=hypertrophic cardiomyopathy genotype positive, phenotype negative; HCM LVH+=hypertrophic cardiomyopathy phenotype positive; LGE=late gadolinium enhancement; LV=left ventricle; LVOT=left ventricular outlet tract; NSVT=non-sustained ventricular tachycardia.

§ n=36.

# n=74.

**Table B.5:** Frequency of types of main sport conducted in 187 study participants.

***Type of sport***	***Frequency***	***Percent***
No exercise history	46	24.6
Cycling	27	14.4
Running	25	13.4
Soccer	15	8.0
Calisthenics	13	7.0
Cross country skiing	10	5.3
Handball	9	4.8
Swimming	9	4.8
Aerobics	5	2.7
Dancing	5	2.7
Field hockey	4	2.1
Basketball	4	2.1
Athletics	2	1.1
Gymnastics	2	1.1
Karate	2	1.1
Pingpong	2	1.1
Archery	1	0.5
Badminton	1	0.5
Figure skating	1	0.5
Speed skating	1	0.5
Alpine skiing	1	0.5
Indoor cycling	1	0.5
Tennis	1	0.5
Total	187	100

## Appendix A

See Fig. A.1, Fig. A.2, Fig. A.3 here.

## Appendix B

See Table B.1, Table B.2, Table B.3, Table B.4, Table B.5 here.

## References

[1] Dejgaard L.A., Haland T.F., Lie O.H., Ribe M., Bjune T., Leren I.S., Berge K.E., Edvardsen T., Haugaa K.H. (2017). Vigorous exercise in patients with hypertrophic cardiomyopathy. Int. J. Cardiol..

